# *Agrobacterium*-Mediated Genetic Transformation of the Edible and Medicinal Cauliflower Mushroom *Sparassis latifolia*

**DOI:** 10.3390/jof12040255

**Published:** 2026-04-01

**Authors:** Wen Cao, Xinyu Zhou, Ruiheng Yang, Yingying Wu, Yan Li, Chenli Zhou, Jianing Wan, Rongping Li, Xiangying Luo, Zhenhui Shen, Dapeng Bao, Lihua Tang, Junjun Shang

**Affiliations:** 1 College of Food Science, Shanghai Ocean University, Shanghai 201306, China; 2 Key Laboratory of Edible Fungi Resources and Utilization (South), Ministry of Agriculture, National Engineering Research Center of Edible Fungi, National R&D Center for Edible Fungi Processing, Institute of Edible Fungi, Shanghai Academy of Agricultural Sciences, Shanghai 201403, China; 3 Institute of Plant Protection, Institute of Mycology, Jilin Agricultural University, Changchun 130118, China; 4 Yunnan Junshijie Biotechnology Ltd., Kunming 650200, China

**Keywords:** *Sparassis latifolia*, *Agrobacterium*-mediated transformation, chlamydospore, hygromycin resistance, carboxin resistance

## Abstract

*Sparassis latifolia* is an edible and medicinal mushroom with significant economic value, now commercially cultivated on a large scale in China. However, current cultivars face challenges, including an extended mycelial growth period and unstable fruiting body yields. Advances in molecular breeding and functional genomics for this species are hindered by the absence of a reliable genetic transformation system. In this study, we first determined that *S. latifolia* is highly sensitive to carboxin and hygromycin, two selective agents commonly used in fungal genetics. We subsequently constructed a novel binary vector, pCbxHyg, harboring a carboxin resistance cassette driven by its native *Pleurotus eryngii* promoter and a hygromycin resistance cassette under the control of the *P. eryngii* Glycerol 3-phosphate dehydrogenase (*GPD*) gene promoter. Initial transformation attempts using *Agrobacterium*-mediated transformation of liquid-cultured mycelial pellets were unsuccessful. During microscopic examination, we discovered that *S. latifolia* mycelia produce abundant asexual chlamydospores. Using these chlamydospores as recipient material, we efficiently and reproducibly obtained transformants with the pCbxHyg vector under both carboxin and hygromycin selection. This method highlights the advantage of using asexual spores of Basidiomycetes as recipients for genetic transformation. PCR analysis confirmed the stable integration of the exogenous resistance genes into the fungal genome. The functionality of the system was further validated by transforming chlamydospores with a vector carrying a β-glucuronidase (*GUS*) reporter gene, whose expression was confirmed via histochemical staining of the resulting transformant mycelia. This work establishes the first successful *Agrobacterium*-mediated genetic transformation system for *S. latifolia*, providing a foundational platform for future gene function studies and molecular breeding efforts.

## 1. Introduction

*Sparassis latifolia* is a tetrapolar heterothallic basidiomycete belonging to the order Polyporales, family Sparassidaceae [1]. Its distinctive, cauliflower-like fruiting body is prized for both its culinary flavor and medicinal properties [2,3,4,5]. The cell wall is rich in bioactive polysaccharides [6,7] that inhibit colon cancer by modulating gut microbiota [8], alleviate side effects of cancer therapies [9], improve intestinal health under high-fat diets [10,11], and exhibit immunomodulatory [12,13,14] and anti-oxidative stress activities [15].

While commercial cultivation of *S. latifolia* has been achieved in China, South Korea, and Japan [16], its industrial development is constrained by an unusually long mycelial growth cycle, high production costs, and inconsistent fruiting body formation [17]. Research efforts to overcome these challenges have included optimizing cultivation techniques [18], cross-breeding [19], and employing genomic and transcriptomic sequencing to elucidate the genetic basis of key agronomic traits [20,21,22,23,24,25]. However, functional validation of candidate genes and molecular breeding require an efficient genetic transformation system, which has not been reported for this species.

Several transformation methods developed for other edible fungi offer valuable precedents. Protoplast transformation mediated by polyethylene glycol (PEG) has been successful in species like *Pleurotus eryngii* [26], *Pleurotus ostreatus* [27] and *Flammulina filiformis* [28]. *Agrobacterium*-mediated transformation (ATMT), which leverages the Ti plasmid to transfer T-DNA into the fungal genome, is a particularly attractive method due to its high efficiency, low transgene copy number, and operational simplicity. It has been widely applied in basidiomycetes such as *Hypsizygus marmoreus* [29], *Lentinula edodes* [30], and *Ganoderma lucidum* [31]. Successful transformation also relies on effective selectable markers, including antibiotic [32] and fungicide resistance genes [33], and reporter genes like Green fluorescent protein (*GFP*) [34] and β-glucuronidase (*GUS*) [35] for monitoring transgene expression.

In this study, we aimed to develop a reliable genetic transformation system for *S. latifolia*. We began by assessing its sensitivity to hygromycin and carboxin. After initial failures with mycelial pellets, we discovered that the mycelia produce chlamydospores. Using these spores as recipients, we successfully established a stable ATMT system, confirmed by molecular analysis and functional *GUS* expression.

## 2. Materials and Methods

### 2.1. Strains and Plasmid Construction

The wild-type *S. latifolia* strain sp11-1 was a commercially cultivated strain from Yunnan Junshijie Biotechnology Ltd. (Kunming, China) and was maintained in our laboratory. *Escherichia coli* DH5α was used for cloning, and *Agrobacterium tumefaciens* strain EHA105 was used for transformation. To construct the binary vector pCbxHyg, the pCAMBIA1300 backbone (CSIRO, Canberra, Australia) was digested with AseI to remove the existing CaMV35S-hygromycin resistance cassette and gel-purified. The carboxin resistance gene (*Cbx^R^*), encoding a mutated succinate dehydrogenase iron-sulfur subunit from *P. eryngii* under the control of its native promoter, was amplified from plasmid pTSdi [26]. The *P. eryngii* Glycerol 3-phosphate dehydrogenase (*GPD*) gene promoter (PeGPDp) and terminator (PeGPDt) were amplified from genomic DNA [36] to drive the hygromycin resistance gene (*Hyg^R^*), which was amplified from pCAMBIA1300. These fragments were assembled into the purified backbone using the ClonExpress™ MultiS One Step Cloning Kit (Vazyme Biotech, Nanjing, China).

### 2.2. Culture Conditions and Preparation of Recipient Material

*S. latifolia* was routinely cultured on YMG medium (1% glucose, 1% malt extract, 0.4% yeast extract, 2% agar for solid media) at 25 °C in the dark.

For mycelial pellets, plugs from a one-month-old colony were inoculated into 100 mL of liquid YMG in 250 mL flasks and incubated at 25 °C with shaking at 150 rpm for 14 days. A 30 mL aliquot was then transferred to 70 mL of fresh medium and cultured for another 14 days.

For chlamydospore isolation, mycelia from plate cultures were gently ground in a 5 mL manual glass cell homogenizer with 2 mL of 0.05% Tween-20. The resulting suspension was filtered through non-woven fabric with a pore size of 22–25 μM to collect the chlamydospores.

### 2.3. Agrobacterium-Mediated Transformation (ATMT)

The pCbxHyg plasmid was introduced into *A. tumefaciens* EHA105 via the freeze–thaw method. A single colony was cultured in LB broth with appropriate antibiotics (rifampicin 50 µg/mL, kanamycin 50 µg/mL) at 28 °C, 200 rpm for 24 h. Bacterial cells were harvested by centrifugation, washed, and resuspended in induction medium (IM: 10 mM K_2_HPO_4_, 10 mM KH_2_PO_4_, 2.5 mM NaCl, 2 mM MgSO_4_•7H_2_O, 0.7 mM CaCl_2_, 9 µM FeSO_4_•7H_2_O, 4 mM (NH_4_)_2_SO_4_, 10 mM glucose, 0.5% glycerol, 0.2 mM acetosyringone, 40 mM MES, pH 5.6) to an OD_600_ of 0.4–0.6 after 6 h of pre-induction.

A 500 µL aliquot of the induced *Agrobacterium* suspension was mixed with an equal volume of either mycelial pellets or a chlamydospore suspension. The mixture was incubated at 28 °C with gentle shaking (80 rpm) for 20 min, then spread onto cellophane-overlaid IM solid plates and co-cultivated at 28 °C for 2 days. After co-cultivation, the recipient material was washed with sterile water and transferred to YMG selection plates containing 300 μg/mL cefotaxime, along with either 2 µg/mL carboxin or 25 µg/mL hygromycin. Plates were incubated at 25 °C until transformant colonies appeared.

### 2.4. Molecular Identification of Transformants

Genomic DNA was extracted from putative transformants that grew stably on selective media. Transformants obtained on hygromycin were screened by PCR with primers HygF-778 (5′-TAAATAGCTGCGCCGATGGT-3′) and HygR-778 (5′-ATTTGTGTACGCCCGACAGT-3′) targeting a 778 bp fragment of Hyg^R^. Transformants from carboxin selection were screened with primers CbxF-564 (5′-TCACATTCCGTCGTTCGTGT-3′) and CbxR-564 (5′-GGCAGCGGAACAAACTCATC-3′) targeting a 564 bp fragment of Cbx^R^. Plasmid pCbxHyg and wild-type *S. latifolia* DNA served as positive and negative controls, respectively.

### 2.5. GUS Reporter Gene Assay

Mycelial pellets from PCR-positive pSdi-GUS transformants and the wild-type strain were immersed in GUS staining solution (Zhongke Tairui Biotech, Beijing, China) and incubated at 37 °C for 24 h. The development of blue coloration was assessed visually.

## 3. Results

### 3.1. Growth Characteristics of S. latifolia

**Figure 1:**
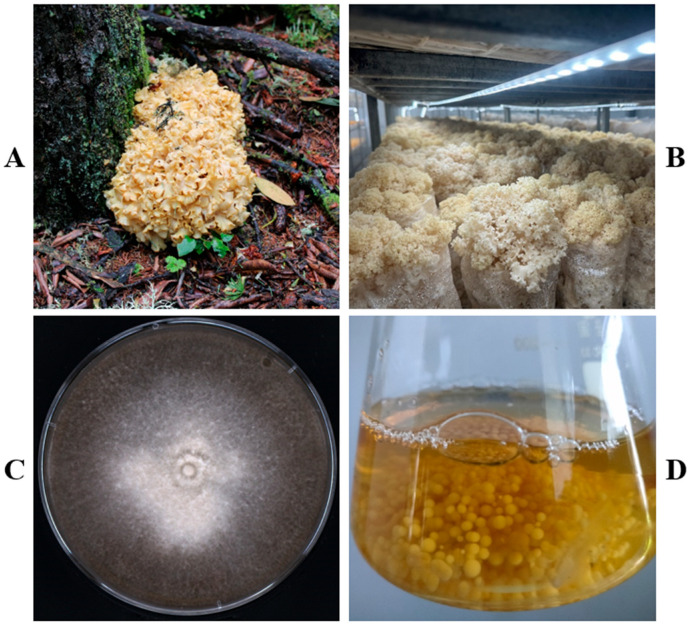
Culture of *S. latifolia*. (**A**) Wild fruiting body. (**B**) Industrially cultivated fruiting body. (**C**) Mycelia grown on solid YMG medium for one month. (**D**) Mycelial pellets obtained from liquid shake culture.

In its natural habitat, *S. latifolia* is a wood-decomposing fungus, typically solitary or cespitose at the base or on stumps of tall trees in coniferous or mixed coniferous–broadleaf forests. Figure 1A shows a wild *S. latifolia* specimen photographed on Haba Snow Mountain, Yunnan Province, China. Under industrial cultivation conditions, the full cultivation cycle of *S. latifolia* from inoculation to harvest is approximately 120 days (Figure 1B), nearly double that of other industrially cultivated mushroom like *P. eryngii*, highlighting the need for genetic improvement.

In laboratory culture, mycelial growth on solid YMG medium is also slow, requiring about 30 days to fully colonize a 9 cm plate (Figure 1C). Liquid shake culture significantly accelerated biomass production, yielding uniform, smooth-surfaced mycelial pellets (2–5 mm in diameter) after 14 days (Figure 1D), which were initially used as transformation recipients.

### 3.2. Sensitivity to Carboxin and Hygromycin

**Figure 2:**
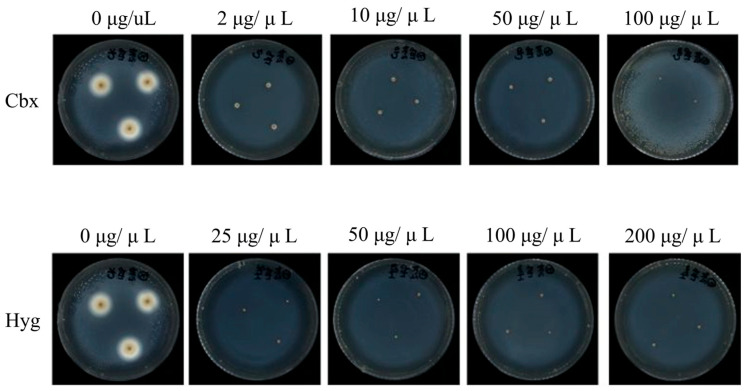
Sensitivity of *S. latifolia* to selective agents. Mycelial growth was completely inhibited on YMG plates containing (up) 2 µg/mL carboxin and (down) 25 µg/mL hygromycin. This sensitivity experiment underwent three biological replicates.

Carboxin, an efficient fungicide, is widely used as a selective agent in the genetic transformation of basidiomycetes. To establish a robust selection system, we determined the minimum inhibitory concentration (MIC) of carboxin and hygromycin for wild-type *S. latifolia*. The concentration ranges of selective agents for testing were determined based on our previous experience with genetic transformation in *H. marmoreus* and *P. eryngii*. Mycelial growth was completely inhibited on medium containing 2 µg/mL carboxin (Figure 2 up) and 25 µg/mL hygromycin (Figure 2 down). We performed three biological replicates of this sensitivity experiment and obtained consistent results. These MIC values were used for all subsequent selection experiments.

### 3.3. Construction of the Binary Vector pCbxHyg

**Figure 3:**
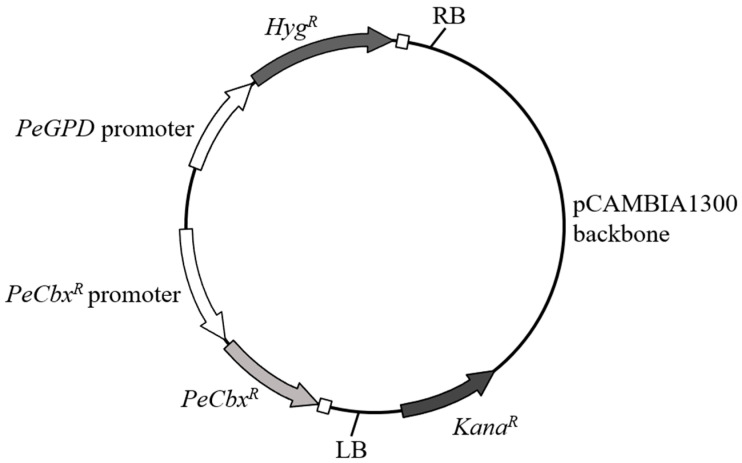
Schematic map of the binary vector pCbxHyg. The T-DNA region (between LB and RB) contains the *Cbx^R^* and *Hyg^R^* expression cassettes. The *P. eryngii*-derived carboxin resistance gene (*Cbx^R^*) was driven by its native promoter, and the hygromycin resistance gene (*Hyg^R^*) was driven by the strong constitutive *P. eryngii* GPD promoter (*PeGPDp*).

To provide flexibility in selection, we constructed the binary vector pCbxHyg (Figure 3). Its T-DNA contains two expression cassettes: a *P. eryngii*-derived carboxin resistance gene (*Cbx^R^*) under the control of its native promoter, and a hygromycin resistance gene (*Hyg^R^*) driven by the strong constitutive *P. eryngii* GPD promoter (*PeGPDp*).

### 3.4. Transformation Attempts Using Mycelial Pellets Are Unsuccessful

**Figure 4:**
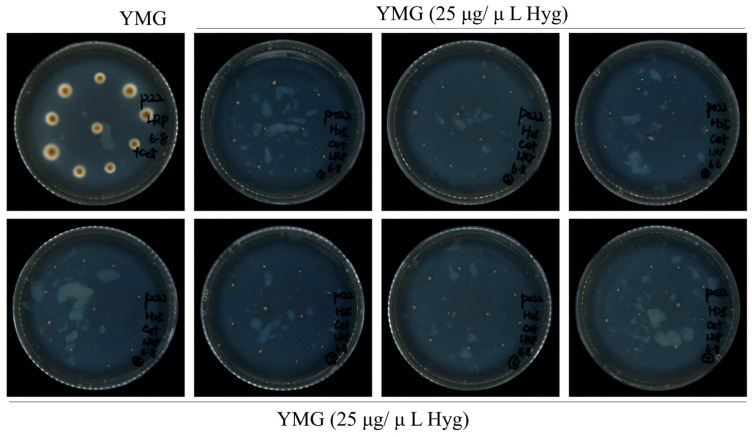
Unsuccessful transformation of mycelial pellets. No resistant colonies regenerated on YMG plates containing 25 µg/mL hygromycin after co-cultivation with *Agrobacterium*.

Despite repeated attempts, no transformants were obtained when using mycelial pellets as recipient material for ATMT with pCbxHyg, under either carboxin or hygromycin selection (Figure 4). Control pellets plated on non-selective medium grew normally, indicating that the co-cultivation process was not inherently lethal. This suggested that the dense, multicellular structure of the pellets might be a physical barrier to efficient *Agrobacterium* infection and T-DNA delivery.

### 3.5. Successful Transformation Using Chlamydospores

**Figure 5:**
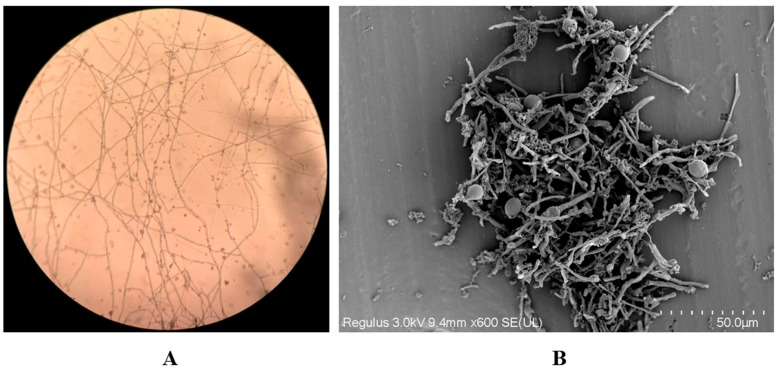
Chlamydospores produced by *S. latifolia*. (**A**) Light micrograph and (**B**) scanning electron micrograph showing chlamydospores associated with mycelia.

Microscopic examination of plate-cultured mycelia revealed the presence of numerous thick-walled, oval structures, which were identified as chlamydospores by scanning electron microscopy (Figure 5). These spores were approximately 5–8 µm in length, with a smooth surface and a diameter larger than that of vegetative hyphae.

**Figure 6:**
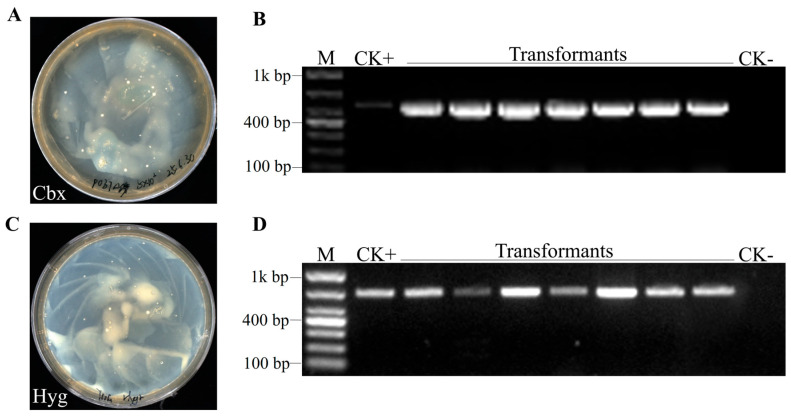
Successful transformation of chlamydospores and molecular confirmation. (**A**,**C**) Transformants obtained on carboxin and hygromycin selection plates. PCR detection of *Cbx^R^* (**B**) and *Hyg^R^* (**D**) in representative transformants. M: marker; CK+: positive control (plasmid DNA); CK-: negative control (wild-type DNA).

When chlamydospores were used as recipients for ATMT with pCbxHyg, transformant colonies appeared on both carboxin and hygromycin selection plates within 3–4 weeks (Figure 6A,C). In three independent genetic transformation experiments using 10^6^ chlamydospores, we obtained 49, 28, and 37 transformants, respectively. Since 10^6^ chlamydospores are readily available, we deemed such transformation efficiency acceptable. We selected 7 transformants each from the carboxin selection plate and the hygromycin selection plate for PCR verification, and the results confirmed the presence of the respective resistance genes in the genomes of all tested transformants, while no amplification was observed in the wild-type control (Figure 6B,D). The selected transformants were subcultured on plates without selection agents. After five rounds of subculture, they were transferred back to plates containing the corresponding selection agents. The results showed that all transformants retained resistance to the respective selection agents, and the introduced foreign genes could still be detected by PCR. This demonstrates that the T-DNA was successfully integrated into the *S. latifolia* genome.

### 3.6. Functional Expression of a GUS Reporter Gene

**Figure 7:**
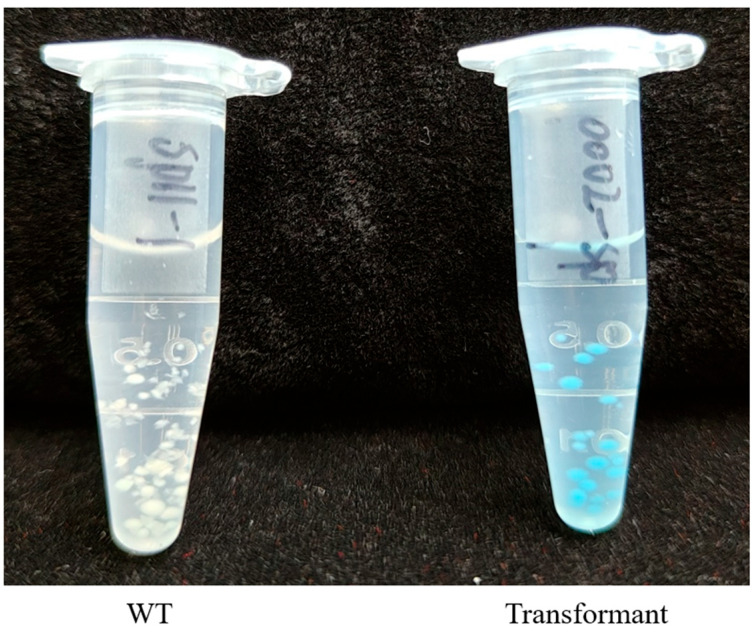
Functional expression of the *GUS* reporter gene. Wild-type mycelial pellets (**left**) show no staining, while pellets from a pSdi-GUS transformant (**right**) stain blue, indicating GUS activity.

To validate that the introduced foreign genes are not only present but also functional, we transformed chlamydospores with the vector pSdi-GUS, which contains a *Cbx^R^* cassette and a *GUS* reporter gene [29]. Carboxin-resistant transformants were readily obtained. Mycelial pellets from these transformants stained intensely blue in a GUS assay, while wild-type pellets showed no color change (Figure 7). This result confirms that the exogenous *GUS* gene is actively transcribed and translated into a functional enzyme in *S. latifolia*.

## 4. Discussion

*Agrobacterium*-mediated transformation has become an important tool for functional genomics research in filamentous fungi due to its advantages, including the ability to efficiently transfer large DNA fragments, typically generate single-copy integrations, and its relatively simple operation. This study reports the first successful establishment of an *Agrobacterium*-mediated genetic transformation system for the economically important mushroom *Sparassis latifolia*. The critical factor enabling this success was the identification and use of asexual chlamydospores as the recipient material, which proved to be highly efficient compared to the use of mycelial pellets.

The failure of transformation with mycelial pellets can be attributed to their complex, three-dimensional structure. The dense network of intertwined, multinucleate hyphae likely presents a physical barrier that prevents uniform attachment of *Agrobacterium* and subsequent T-DNA delivery to a sufficient number of competent cells. In contrast, chlamydospores, as unicellular, thick-walled structures poised for germination, provide a uniform and accessible target. Their cell wall remodeling during germination may facilitate T-DNA integration, highlighting a principle common in fungal transformation: the superiority of using single cells over multicellular tissues as recipients. In *P. eryngii*, protoplasts were used as recipients to establish a PEG-mediated genetic transformation system [26], and in *H. marmoreus*, arthroconidia were used as recipients to establish an *Agrobacterium*-mediated genetic transformation system [29], both demonstrating the advantage of using single-cell recipients.

The successful expression of heterologous genetic elements from *P. eryngii* in *S. latifolia* is another notable finding. The *P. eryngii* GPD promoter efficiently drove *Hyg^R^* expression, and the *Cbx^R^* gene also functioned effectively. This suggests a high degree of functional conservation in core regulatory and metabolic machinery within basidiomycetes, despite their phylogenetic distance. This cross-species functionality has practical implications, as it allows researchers to leverage well-characterized genetic tools from model species without the need to clone and test native sequences from every new target fungus, thereby accelerating research in non-model systems.

In conclusion, the chlamydospore-based ATMT system developed here is robust, efficient, and reproducible. It enables both stable integration and functional expression of foreign genes in *S. latifolia*. This platform will be instrumental for future functional genomics studies, such as gene knockout and overexpression, to dissect the molecular basis of its long life cycle and other agronomic traits. Ultimately, this system paves the way for the molecular breeding of improved *S. latifolia* cultivars with shorter cultivation cycles, higher yields, and enhanced traits.

## Data Availability

The original contributions presented in this study are included in the article. Further inquiries can be directed to the corresponding authors.
